# New Human Leukocyte Antigen (HLA) Antibody Formation and Creatinine Elevation With Abaloparatide in Kidney Transplant Recipient

**DOI:** 10.1002/jbm4.10814

**Published:** 2023-09-21

**Authors:** Christine M. Swanson, Kelly Krohn, Alexander Wiseman, Micol S. Rothman

**Affiliations:** ^1^ Division of Endocrinology, Metabolism and Diabetes University of Colorado Anschutz Medical Campus Aurora CO USA; ^2^ Eastern Colorado VA Geriatric, Research, Education, and Clinical Center (GRECC) Aurora CO USA; ^3^ The Core Institute Phoenix AZ USA; ^4^ Division of Nephrology Centura Transplant Denver CO USA

**Keywords:** ABALOPARATIDE, IMMUNOGENICITY, KIDNEY TRANSPLANT, OSTEOPOROSIS, TERIPARATIDE

## Abstract

A 39‐year‐old female with a history of kidney transplant presented to the endocrinology clinic for osteoporosis evaluation after sustaining an ankle fracture from a fall. Her kidney transplant regimen (mycophenolate mofetil 360 mg twice a day, tacrolimus 0.5 mg every morning and 0.5–1 mg every evening, prednisone 5 mg/day) and baseline creatinine (1.0–1.2 mg/dL) had been stable for several years. After an appropriate secondary workup, she was started on abaloparatide 80 μg subcutaneous daily injections for osteoporosis. She had a good initial biochemical response to therapy. However, 5 months after abaloparatide initiation she was found to have a new elevation in serum creatinine (1.17 to 1.69 mg/dL) despite stable serum tacrolimus trough levels, and two new human leukocyte antigen (HLA) antibodies (anti‐HLA antibodies detected to Cw7 and DP28). Abaloparatide was stopped due to concern for immunogenicity. There was no evidence of rejection on kidney biopsy and she was restabilized on her transplant regimen with a new baseline creatinine of 1.3–1.6 mg/dL. The patient was subsequently started on teriparatide 20 μg daily subcutaneous injections for 2 years with good biochemical response, significant improvement in bone mineral density, and stable transplant regimen without additional signs of immunogenicity or rejection. This is the first case report to raise concern about immunogenicity with abaloparatide in solid organ transplant recipients. © 2023 The Authors. *JBMR Plus* published by Wiley Periodicals LLC on behalf of American Society for Bone and Mineral Research.

## Case Report

A 39‐year‐old female with a history of ulcerative colitis, premature ovarian insufficiency at age 37 years, pulmonary embolism, and end‐stage renal disease secondary to biopsy‐proven immunoglobulin A (IgA) nephropathy status post living donor–related kidney transplant in 2006 on chronic prednisone presented to the Endocrinology clinic for evaluation of low bone mineral density (BMD) and fracture. Osteoporosis was diagnosed in 2007 at 27 years old on dual‐energy X‐ray absorptiometry (DXA) that was performed due to her history of transplant and chronic prednisone use (lowest *T*‐score −3.4, lowest *Z*‐score −2.6). She took risedronate for 5 years (2007–2012) and subsequently sustained a left ankle avulsion fracture when she missed one step walking down a staircase in 2019. Her mother had osteoporosis. She had no history of radiation therapy or kidney stones.

She was diagnosed with IgA nephropathy with kidney biopsy in 2005 and was on hemodialysis for 5 months. She received an human leukocyte antigen (HLA) 5 antigen mismatched, living donor–related kidney transplant in 2006. One month after transplant she had an episode of Banff 1A rejection for which she received thymoglobulin with high dose methylprednisolone (Solu‐Medrol) for 6 weeks with clinical resolution. The prednisone dose was titrated down to 10 mg/day for approximately 5 years. She was stable on prednisone 5 mg/day from 2011 until presentation in 2019. Her anti‐rejection transplant medication regimen also included mycophenolate mofetil 360 mg twice a day and tacrolimus (0.5 mg every morning, 0.5–1 mg every evening). Her tacrolimus goal was 4–7 ng/mL and her tacrolimus trough level ranged from 6.0 to 10.5 ng/mL from 2015 through early 2020. Antibodies were monitored annually/semiannually with no evidence of donor specific antibodies in 2015, 2017, 2018, 2019, or February 2020. Her transplant regimen with a baseline creatinine of 1.0–1.2 mg/dL had been stable for approximately 14 years.

Menarche occurred at 14 years old with subsequently irregular periods attributed to polycystic ovarian syndrome. She experienced a pulmonary embolism in 2008 while on oral contraceptive pills and previously completed a year of warfarin (Coumadin) therapy. No underlying clotting disorder was identified on Hematology evaluation. Hematology recommended chronic anticoagulation if exogenous hormones were ever restarted. She had a left oophorectomy in 2010 for a large ovarian cyst and a right cystectomy and partial salpingectomy in 2012 for an incidentally noted large right ovarian cyst. Her last menstrual period was in January 2017, at the age of 37 years. Premature ovarian insufficiency was attributed to insufficient ovarian tissue after the surgical procedures.

She was diagnosed with ulcerative colitis based on colonoscopy in 2016 and received a 3‐month course of budesonide. In 2016 she also had gastroparesis (attributed to mycophenolate mofetil [Cellcept] therapy), resulting in a period of low body mass index (BMI) (16 kg/m^2^) for approximately 18 months in 2016–2018. Her BMI at presentation in 2019 was 19.6 kg/m^2^.

She was started on calcium and vitamin D supplementation when prednisone was initiated in 2006. Despite this, she had hypocalciuria during evaluation in 2019 (24‐hour urine calcium = 72 mg/day; Table 1) due to lactose intolerance and historically poor dietary calcium intake. Celiac panel was negative and urine calcium improved with increased intake of nondairy sources of dietary calcium (24‐hour urine calcium = 108 mg/day in 2020 prior to medication initiation; Table 1). She was a lifelong nonsmoker with minimal intake of alcohol and caffeine and was physically active. She had no steroid exposure prior to her kidney transplant. Bone turnover markers were elevated (Table 1). No additional secondary causes of low BMD were identified on laboratory evaluation performed in 2019 including normal albumin‐adjusted total serum calcium, serum phosphorous, and serum alkaline phosphatase with a parathyroid hormone (PTH) of 58 pg/mL (Table 1). Given the brief duration of remote hemodialysis, no evidence of secondary hyperparathyroidism, currently normal kidney function, and elevated bone turnover markers without clinical concern for adynamic bone disease, bone biopsy was not performed at the time of evaluation.

**Table 1:** Pertinent Laboratory Results From Evaluation for Secondary Causes of Osteoporosis in December 2019, Abaloparatide Initiation in February 2020, Identification of Immunogenicity in July 2020, and Subsequent Two‐Year Course of Teriparatide Starting in August 2020

Laboratory results	1/2018	2/2019	7/2019	12/2019	2/2020	3/2020	7/2020	11/2020	2/2021	3/2021	9/2021
Ca (mg/dL), normal 8.6–10.3	9.4	9.5	9.2	9.4		9.5			10.1	9.4	9.4
Albumin (g/dL), normal 3.5–5.7	4.5	4.6	4.5	4.3		4.2					
Phosphorous (mg/dL), normal 2.5–5.0	2.9	3.4	2.9	4.8		4.2					
25OHD (ng/mL)	71	34		43					35		46
PTH (pg/mL), normal 12–88	71	63		58					57.7		34.3
Cr (mg/dL), normal 0.6–1.20	1.23	1.02	1.15	1.23		1.17			1.55	1.41	1.33
Alkaline phosphatase (U/L), normal 39–117	101	134	148	103		94					
TSH (μIU/mL), normal 0.45–5.33				1.03						1.46	
Mg (mg/dL), normal 1.6–2.6		1.9	1.9								
CBC				Normal							
SPEP				Normal							
Celiac panel				Negative							
24‐hour urine calcium (mg/day)				72	108						
24‐hour urine creatinine (mg/day)				834	897						
CTX (pg/mL), premenopausal normal 136–689 pg/mL				722							
BSAP (μg/L), premenopausal normal 4.5–16.9 μg/L				21.8							
P1NP (μg/L), premenopausal normal 20–101 μg/L				122		185		99			
Tacrolimus trough level (ng/mL)	7.2	9.8	6.1	6.2	6.9	5.9	4.4	4.9	6.4	4.9	6.5
Tacrolimus daily dose (mg/day)	1.5	1.5	1.5	1.5	1.5	1.5	1.5	2.0	2.0	2.0	2.0

Abbreviation: BSAP = bone‐specific alkaline phosphatase; CBC = complete blood count; SPEP = serum protein electrophoresis; TSH = thyroid‐stimulating hormone.

Given the patient's past medical history, she desired an aggressive approach to treating her osteoporosis to avoid additional fractures and maintain her mobility and independence. She previously received 5 years of bisphosphonate therapy, most recently in 2012. Her young age required a long‐term treatment approach that would maximize therapeutic benefit to decrease fracture risk now and in the future, without committing her to continuous lifelong therapy. Given the complexity of her case, treatment decisions were discussed, in depth, with an online community of medical providers specializing in metabolic bone disease (Bone Health TeleECHO),^(^ ^1^, ^2^ ^)^ the patient, and her family. She had contraindications and/or significant risks with several US Food and Drug Administration (FDA)‐approved osteoporosis treatments including estrogen/raloxifene (due to history of pulmonary embolism and requirement for concurrent anticoagulation), oral bisphosphonates (due to history of prior gastroparesis, ulcerative colitis, and prior 5‐year course), denosumab (dosing profile would commit her to long‐term therapy, which was less desirable in a young individual), and romosozumab (cardiovascular concern given history of pulmonary embolism). Therefore, initiation of anabolic therapy followed by a single consolidation dose of zoledronate was determined to be the best treatment course for her, particularly with data supporting this sequential therapy approach.^(^ ^3^ ^)^ Abaloparatide (ie, Tymlos) was chosen over teriparatide (ie, Forteo) due to the lack of refrigeration requirement.

She initiated abaloparatide 80 μg daily in February 2020. Laboratory tests done in March 2020 showed normal albumin‐adjusted calcium and an excellent biochemical response to therapy^(^ ^4^ ^)^ with the non‐renally‐cleared bone formation marker, procollagen type 1 N‐terminal propeptide (P1NP; Table 1), increasing from 122 μg/L pretreatment to 185 μg/L on treatment (premenopausal normal range 20–101 μg/L). She developed fatigue, headaches, and palpitations for 1–2 hours after each abaloparatide injection. These symptoms were considered to be consistent with typical vasodilation side effects of the medication. Although the duration of these symptoms improved over time, a trial of teriparatide was recommended to see if this improved her symptoms. In July 2020, before she could change from abaloparatide to teriparatide, she was evaluated by her Transplant Nephrology team for an increase in serum creatinine from 1.17 to 1.69, despite stable serum tacrolimus levels, and new HLA antibody formation (HLA‐Cw7, mean fluorescent intensity 1930, HLA‐DP28, mean fluorescent intensity 1020) (Table 1). She stopped the abaloparatide on July 17, 2020. There was no evidence of rejection on kidney biopsy. Specifically, there was no interstitial inflammation and no evidence of antibody‐mediated injury. She was restabilized on tacrolimus with a new baseline creatinine of 1.3–1.6 mg/dL (previous baseline 1.0–1.2 mg/dL).

**Fig. 1:**
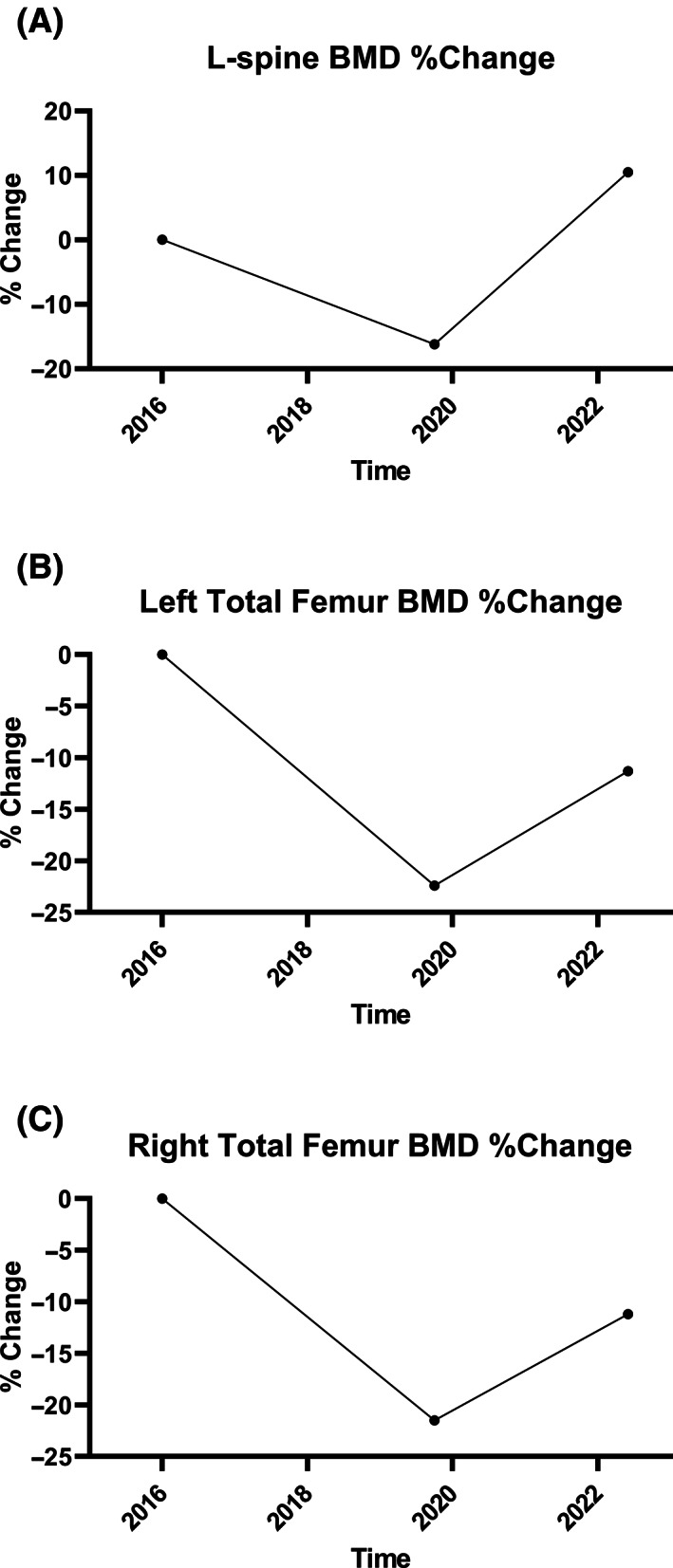
Percent change in bone mineral density (BMD) compared to prior exam at the (*A*) lumbar spine, (*B*) left total femur, and (*C*) right total femur. BMD increased 26.7%, 11.1%, and 10.3%, respectively from 2019 to 2022 with interim teriparatide daily injections.

After an informed discussion and shared decision making with the patient, her family, her transplant nephrology team, and the online community of bone health experts (Bone Health TeleECHO),^(^ ^1^, ^2^ ^)^ the patient started teriparatide 20 μg daily in August 2020. Calcium and creatinine levels remained stable after teriparatide initiation and throughout the subsequent 2‐year course. She had an excellent response to teriparatide based on P1NP levels (Table 1) and BMD (Fig. 1), with no subsequent fractures. Subsequent testing for HLA antibodies were negative.

## Discussion

There are no reports in the literature of abaloparatide being associated with immunogenicity in solid organ transplant recipients. This case report cannot establish a cause‐and‐effect relationship. However, the prior duration of transplant regimen stability and the timing of abaloparatide initiation relative to new HLA antibody formation with no other interim clinical events increases suspicion that abaloparatide played a causative role. De novo donor‐specific antibodies occur in ~10%–15% of patients by 5 years posttransplant and it is unusual for patients to develop HLA antibodies late after transplant in the setting of appropriate immunosuppression.^(^ ^5^, ^6^, ^7^ ^)^ At the time of the patient's transplant in 2006, HLA typing at the Cw and DP loci was not a part of routine clinical practice so it cannot be determined if the HLA antibodies she developed were donor specific versus non‐donor specific. It is unclear what role, if any, the patient's history of autoimmune disease played in the development of new HLA antibodies after abaloparatide initiation.

There are two anabolic medications related to PTH that are FDA‐approved to treat postmenopausal osteoporosis, abaloparatide and teriparatide. Abaloparatide is a synthetic peptide analog of PTH‐related protein (PTH‐rp) that transiently stimulates the parathyroid hormone receptor type 1 (PTHR1) resulting in an anabolic skeletal response.^(^ ^8^, ^9^ ^)^ In postmenopausal women, abaloparatide therapy causes early increases in bone turnover markers (more so for P1NP than the resorption marker C‐telopeptide of type I collagen [CTX]), corresponding with improved BMD at the lumbar spine and total hip, and a decrease in vertebral, clinical, and major osteoporotic fractures compared to placebo.^(^ ^8^, ^10^ ^)^ In a Phase II study, 12% of patients receiving abaloparatide developed anti‐abaloparatide antibodies after 24 weeks but no immune‐related events were reported.^(^ ^8^ ^)^ Antibody development did not confer an increased risk of adverse events, although one patient developed abaloparatide‐neutralizing activity that did not appear to attenuate medication efficacy.^(^ ^8^ ^)^

The PTH‐analog, teriparatide, has the same first 34‐amino acid sequence as endogenous PTH and also stimulates the PTHR1 receptor to generate an anabolic skeletal response.^(^ ^11^ ^)^ In postmenopausal women, teriparatide stimulates bone turnover markers, increases BMD at the lumbar spine and femoral neck, and decreases risk of vertebral and nonvertebral fractures.^(^ ^11^ ^)^ In the Neer and colleagues^(^ ^11^ ^)^ study, PTH antibodies developed in one woman in the placebo group (<1%), 15 women receiving the FDA‐approved 20 μg/day dose (3%), and 44 women receiving the 40 μg/day dose (8%) but with no discernible effect on any outcome measures.

The trials of PTH‐based anabolic osteoporosis medications referenced above were performed in healthy postmenopausal women, not in solid organ transplant recipients. There are no large, long‐term randomized controlled trials of anabolic medications in kidney transplant patients. However, teriparatide has been used in 15 hypoparathyroid patients with a history of kidney transplant with no episodes of rejection.^(^ ^12^, ^13^ ^)^ In a 6‐month, double‐blind randomized trial by Cejka and colleagues^(^ ^14^ ^)^ in 26 transplant recipients, teriparatide stabilized femoral neck BMD in the first 6 months after kidney transplant (compared to BMD loss in the placebo group), and there were numerically fewer episodes of rejection in the teriparatide group compared to those who received placebo. Significant improvements in bone formation on transiliac bone biopsy was observed in a heart transplant recipient with chronic kidney disease who was initiated on teriparatide for low bone turnover disease with fracture.^(^ ^15^ ^)^ Based on PubMed searches performed in 2020, June 2022, and June 2023, no articles have been published on the use of abaloparatide in kidney transplant recipients. Because abaloparatide is a *synthetic* peptide, it may be more likely to trigger an immune response than the endogenous peptide teriparatide. This could be consistent with the higher percentage of women developing antibodies in the initial abaloparatide studies compared to the initial teriparatide study.^(^ ^8^, ^11^ ^)^ However, because these trials were conducted in different study populations, there may have been additional factors contributing to the numerical imbalance.

Bisphosphonate medications are the most well‐studied osteoporosis medications in kidney transplant patients.^(^ ^16^ ^)^ A recent Cochrane review found that bisphosphonate therapy *may decrease* acute graft rejection.^(^ ^16^ ^)^ In the long‐term, bisphosphonates may improve graft survival in kidney transplant recipients, possibly by preventing acute and chronic rejection.^(^ ^17^ ^)^ The Cochrane review also concluded that due to small sample sizes it is uncertain whether any other osteoporosis treatment (including “synthetic PTH”) impacts graft rejection.^(^ ^16^ ^)^

In conclusion, this is the first report of a temporal relationship between abaloparatide initiation and the development of new low‐level HLA antibody formation in a kidney transplant recipient who subsequently did well on teriparatide. Additional studies are needed to assess the safety and efficacy of anabolic medications in solid organ transplant recipients.

## Author Contributions

**Christine M. Swanson:** Conceptualization; data curation; investigation; writing – original draft; writing – review and editing. **Kelly Krohn:** Writing – review and editing. **Alexander Wiseman:** Data curation; investigation; writing – review and editing. **Micol S. Rothman:** Conceptualization; project administration; writing – review and editing.

## Disclosures

CMS, MSR: None. KK: Speaker for Radius. AW: Consultant Transplant Genomics, Nephrosant, Sanofi, Veloxis and Speaker. Bureau Transplant Genomics, Sanofi, Veloxis.

## Data Availability

Deidentified data can be made available upon reasonable request, as governed by Health Insurance Portability and Accountability Act (HIPAA) regulations.

## References

[1] Lewiecki EM , Jackson A 3rd , Lake AF , et al. Bone health TeleECHO: a force multiplier to improve the care of skeletal diseases in underserved communities. Curr Osteoporos Rep. 2019;17(6):474–482. 31713181 10.1007/s11914-019-00543-9

[2] Rothman MS , Olenginski TP , Stanciu I , Krohn K , Lewiecki EM . Lessons learned with Bone Health TeleECHO: making treatment decisions when guidelines conflict. Osteoporos Int. 2019;30(12):2401–2406. 31471665 10.1007/s00198-019-05147-8

[3] Cosman F . Anabolic and antiresorptive therapy for osteoporosis: combination and sequential approaches. Curr Osteoporos Rep. 2014;12(4):385–395. 25341476 10.1007/s11914-014-0237-9

[4] Glover SJ , Eastell R , McCloskey EV , et al. Rapid and robust response of biochemical markers of bone formation to teriparatide therapy. Bone. 2009;45(6):1053–1058. 19679211 10.1016/j.bone.2009.07.091

[5] Davis S , Wiebe C , Campbell K , et al. Adequate tacrolimus exposure modulates the impact of HLA class II molecular mismatch: a validation study in an American cohort. Am J Transplant. 2021;21(1):322–328. 32888256 10.1111/ajt.16290 PMC7821185

[6] Wiebe C , Gibson IW , Blydt‐Hansen TD , et al. Evolution and clinical pathologic correlations of de novo donor‐specific HLA antibody post kidney transplant. Am J Transplant. 2012;12(5):1157–1167. 22429309 10.1111/j.1600-6143.2012.04013.x

[7] Wiebe C , Rush DN , Nevins TE , et al. Class II Eplet mismatch modulates tacrolimus trough levels required to prevent donor‐specific antibody development. J Am Soc Nephrol. 2017;28(11):3353–3362. 28729289 10.1681/ASN.2017030287 PMC5661295

[8] Leder BZ , O'Dea LS , Zanchetta JR , et al. Effects of abaloparatide, a human parathyroid hormone‐related peptide analog, on bone mineral density in postmenopausal women with osteoporosis. J Clin Endocrinol Metab. 2015;100(2):697–706. 25393645 10.1210/jc.2014-3718

[9] Hattersley G , Dean T , Corbin BA , Bahar H , Gardella TJ . Binding selectivity of Abaloparatide for PTH‐Type‐1‐receptor conformations and effects on downstream signaling. Endocrinology. 2016;157(1):141–149. 26562265 10.1210/en.2015-1726 PMC4701881

[10] Miller PD , Hattersley G , Riis BJ , et al. Effect of Abaloparatide vs placebo on new vertebral fractures in postmenopausal women with osteoporosis: a randomized clinical trial. JAMA. 2016;316(7):722–733. 27533157 10.1001/jama.2016.11136

[11] Neer RM , Arnaud CD , Zanchetta JR , et al. Effect of parathyroid hormone (1–34) on fractures and bone mineral density in postmenopausal women with osteoporosis. N Engl J Med. 2001;344(19):1434–1441. 11346808 10.1056/NEJM200105103441904

[12] Hod T , Riella LV , Chandraker A . Recombinant PTH therapy for severe hypoparathyroidism after kidney transplantation in pre‐transplant parathyroidectomized patients: review of the literature and a case report. Clin Transplant. 2015;29(11):951–957. 26331695 10.1111/ctr.12622

[13] Nogueira EL , Costa AC , Santana A , et al. Teriparatide efficacy in the treatment of severe hypocalcemia after kidney transplantation in parathyroidectomized patients: a series of five case reports. Transplantation. 2011;92(3):316–320. 21694663 10.1097/TP.0b013e3182247b98

[14] Cejka D , Benesch T , Krestan C , et al. Effect of teriparatide on early bone loss after kidney transplantation. Am J Transplant. 2008;8(9):1864–1870. 18786230 10.1111/j.1600-6143.2008.02327.x

[15] Fahrleitner‐Pammer A , Wagner D , Krisper P , Amrein K , Dimai H . Teriparatide treatment in a heart transplant patient with a chronic kidney disease and a low‐turnover bone disease: a case report. Osteoporosis Int. 2017;28(3):1149–1152. 10.1007/s00198-016-3858-2 PMC5406430 27988794

[16] Palmer SC , Chung EY , McGregor DO , Bachmann F , Strippoli GF . Interventions for preventing bone disease in kidney transplant recipients. Cochrane Database Syst Rev. 2019;10:CD005015. 31637698 10.1002/14651858.CD005015.pub4 PMC6803293

[17] Song SH , Choi HY , Kim HY , et al. Effects of bisphosphonates on long‐term kidney transplantation outcomes. Nephrol Dial Transplant. 2021;36(4):722–729. 33367861 10.1093/ndt/gfaa371

